# Lessons from Sicilian Centenarians for Anti-Ageing Medicine. The Oxi-Inflammatory Status

**DOI:** 10.37825/2239-9754.1036

**Published:** 2022-09-26

**Authors:** Calogero Caruso, Giulia Accardi, Anna Aiello, Anna Calabrò, Mattia E. Ligotti, Giuseppina Candore

**Affiliations:** Laboratory of Immunopathology and Immunosenescence, Department of Biomedicine, Neuroscience and Advanced Diagnostics, University of Palermo, Palermo, Italy

**Keywords:** Centenarians, Inflammation, Longevity, Oxidative stress

## Abstract

Population ageing is a great achievement of humanity, but it also represents a challenge that the Western world is currently facing, as ageing is associated with increased susceptibility to age-related inflammatory diseases. Therefore, it is necessary to fully understand the mechanisms of healthy ageing to prevent the harmful aspects of ageing. The study of long living individuals (LLIs) is a great model for trying to achieve this goal. Accordingly, the oxy-inflammatory status of Sicilian LLIs was reviewed in the present paper. Based on the reported data, anti-inflammatory and anti-oxidative stress strategies have been discussed, useful for delaying or avoiding the onset of age-related diseases, thus favouring a healthy ageing process.

## 1. Introduction

People around the world are living longer. In addition, the pace of ageing of the world population is also increasing [1]. The increasing mean lifespan of the population is a big success of humanity, but also poses a challenge that industrialized countries are currently facing because the increase in lifespan does not coincide with the increase in the duration of health, health-span, *i.e.*, the period of life free from serious chronic diseases and disabilities [2]. Therefore, improving the quality of life of oldest people is becoming a priority due to the continuous increase in the number of this population who is at risk of frailty. This makes the studies of the processes involved in healthy ageing and longevity of great importance. The identification of the factors that predispose to a long and healthy life is therefore of enormous interest for translational medicine. This means understanding why some individuals, namely the centenarians, have escaped neonatal mortality, infectious diseases in the pre-antibiotic era, and the fatal outcomes of age-related diseases, thus living more than 100 years. The knowledge born from this approach could allow modulating the ageing rate by providing valuable information on lifestyle to achieve healthy ageing [3–5].

In the last twelve years, we have surveyed the population of some Sicilian small towns and villages, characterized by a high rate of centenarians to investigate the mechanisms involved in longevity [6–10]. Considering the key role played by inflammation and oxidative stress in mechanisms of age-related diseases [11], we report data related to the oxi-inflammatory status of a homogeneous population of Sicilian centenarians and nonagenarians (log-living individuals, LLIs) studied together with young people, adults, and older adults to have an adequate and matched number of controls. Data are summarized and discussed according to the available literature, for their possible implication for the prevention and/or treatment of age-related diseases.

## 2. Oxi-inflammatory status of Sicilian LLIs

**Fig. 1:**
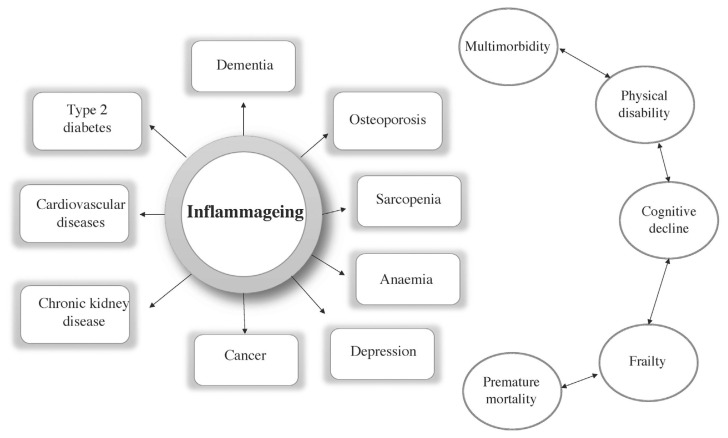
Cardiovascular diseases, chronic kidney disease, cancer, depression, anaemia, sarcopenia, osteoporosis, and dementia are shown in the figure as example of inflammatory age-related diseases, since inflamm-ageing contributes to the development of these diseases in older people. Moreover, elevated blood levels of pro-inflammatory markers are a powerful risk facor for multimorbidity that, with inflamm-ageing, is a strong risk factor for physical and cognitive disability, frailty, and premature mortality. References in the text.

Inflammation is defined as a localized response with systemic consequences, elicited by trauma or infection, which helps to destroy, reduce, or sequester both the harmful agent and the wounded tissue, so supporting survival by fighting off pathogenic microbes and healing injuries. Therefore, the inflammation process is not *per se* a negative phenomenon; it is the response of the innate immune system to pathogenic viruses or bacteria. However, older organisms tend to develop a pro-inflammatory status characterized by high levels of pro-inflammatory markers in cells, tissues, and blood, a condition called inflamm-ageing. In fact, ageing is accompanied by chronic low grade inflammatory state. This chronic inflammation is instrumental in driving the ageing process in general, and contributes to the development of age-related diseases. In older people, the tissues have high levels of proinflammatory cytokines, which are known to interfere with anabolic signalling, thus contributing to the development of sarcopenia. During the lifehistory, low-grade inflammation, develops gradually and contributes to the pathogenesis of a range of age-related diseases as cardiovascular diseases (CDVs), and Type-2 diabetes (T2D), cancer, and neurodegeneration as well as frailty and sarcopenia (a far from exhaustive list is shown in Fig. 1). Therefore, this backdrop of low-grade inflammation contributes significantly to mortality risk of older people and has several sources, as discussed below [12–15].

Oxidative stress is an oxidative condition, resulting from an imbalance between oxidant and antioxidant factors in favour of the former ones, capable of damaging DNA, lipids, and proteins with consequent tissue damage and acceleration of ageing process. With advancing age, the concentrations of free radical increase because of the age-related functional decline in the electron transport chain and changes in the gene expression profile as well as by the decline of antioxidant systems [16]. The excess of free radicals interacts with lipids, proteins, and DNA, altering the normal physiological functions of cells. This ability allows to understand why oxidative stress is involved in the pathophysiological mechanisms of age-related diseases. In addition, the oxidative stress is thought to be closely related to inflamm-ageing [17]. The increase of free radicals is correlated to damage-associated molecular pattern (DAMPs) release, endogenous nuclear or cytosolic molecules released from injured and dying cells. DAMPs are able to activate inflammasome, through specific receptors, promoting the production of pro-inflammatory cytokines. In turn, these cytokines activate inflammatory cells, further potentiating free radicals production, in a vicious cycle [11].

More in general, cellular injury can release DAMPs activating innate immunity. So, cell debris caused by inappropriate cell destruction and clearance during the ageing process can trigger the innate immune system, which sets the scene for persistent inflammation. Cell debris accumulate with age because of both increased production and impaired elimination by defective autophagy [18,19].

However, the most important causes of inflammaageing are represented by cellular senescence and pathogen burden, both linked to the ageing of immune system, *i.e.*, immunosenescence; in turn, inflamm-ageing contributes to immunosenescence [20,21].

Cells are driven into a senescent, non-dividing state by several factors, including telomere shortening, DNA damage, and inflammatory cytokines. These all result in the activation of transcription factor p53 which is involved in a variety of processes including DNA repair and apoptosis, but most notably results in expression of cyclin-dependent kinase inhibitor p21, which together with activation of p16, is the major pathways in the induction of senescence. The immune system clears away these damaged cells; however, in older people immunosenescence is responsible for a less efficiency of the clearing of these cells declines, hence increasing numbers linger in tissues and secrete an inflammatory cocktail of cytokines known as the senescence-associated secretory phenotype (SASP) [21,22].

It has been suggested that the reduction in life-time exposure to infectious diseases and other sources of inflammation contributes to the historical decline in old-age mortality, strengthening the suggestion that long-life pathogen burden is a very important factor for age-related inflammation. Persistent antigenic challenges lead to a poor response to newly encountered microbial antigens, as well as to a shift in the immune system toward an inflammatory T helper profile. Late-stage memory effector T-cells contribute to the pro-inflammatory state in older people as producers of pro-inflammatory cytokines. In addition, the long-term chronic microbial burden induces progressive activation of macrophages, hence contributing to the chronic state of low-grade inflammation. Accordingly, some studies have linked an individual exposure to past infection to levels of chronic inflammation and to increased risk of heart attack, stroke, and cancer [14,15,20,23].

On the other hand, bacteria have developed an intimate relationship with animals, colonizing specific body sites at the interface with the body exterior and invaginations, hence constituting an integrated meta-organism with functional integration conferring significant advantages to animals and bacteria. The immune system of the host coevolved with the microbiota to develop complex mechanisms to recognize and destroy invading microbes, while preserving its own bacteria. However, the oral and gut mucosa barriers that protect against bacterial invasion begin to both decline in effectiveness and break down as human beings age. Periodontal disease has been shown to shown to contribute to inflamm-ageing by generating chronic, low-grade inflammation. In the gut, the microbiome shows an increasing decline of diversity with age. Composition and diversity of the microbiota change according to development and ageing and contribute to health and fitness by modulating the immune system response and inflamm-ageing and vice versa [14,24–26]. Bian et al. [27], collected and examined the gut microbiota of a cross-sectional cohort of more than 1000 very healthy Chinese individuals who spanned ages from 3 to over 100 years, showing that the healthiest aged people had gut microbiomes like much younger people; in other words, they maintained diversity and thus lower levels of inflammation.

Closely related to inflamm-ageing is metaflammation, the low-grade, chronic inflammation orchestrated by metabolic cells in response to excess nutrients and energy. An excess of macronutrients introduced with the diet determines an increased circulation of fatty acids and obesity. That leads to the stimulation of macrophages and the same adipocytes, triggering signal transduction pathways involved in the pro-inflammatory status. In fact, in subjects affected by visceral obesity increased levels of pro-inflammatory cytokines and decreased adiponectin levels are observed. That triggers an increased production of free radicals [28]. Thus, proinflammatory activation of macrophages in metabolic tissues is critically important in the induction of obesity-induced metaflammation and it has been demonstrated that the soluble mannose receptor plays a direct functional role in both macrophage activation and metaflammation [29]. In turn, the pro-inflammatory status induced by metaflammation as well as inflamm-ageing leads to cellular damage and senescence, so worsening inflamm-ageing [28].

In a recent multi-cohort study [30], then, using data from several European countries, it was observed that a disadvantageous socioeconomic status at any stage of life was associated with an increase in inflammation assessed using serum levels of C-reactive protein (CRP), an acute phase reactant that responds rapidly to tissue injury, infection, and inflammation. In general, known behavioural factors (alcohol consumption, smoking habit, and sedentary lifestyle) and body mass index (BMI) explained in part, but not completely, the relationship between socioeconomic status and inflammation. A subsequent analysis indicated that low-educated participants had higher CRP levels regardless of socioeconomic status in early life or adulthood, as well as behavioural factors and BMI. The persistence of a significant association between low level of education and a high level of CRP in adulthood indicate that the level of education may be an upstream risk factor for high inflammation. These findings suggest that there are other pathways through which socioeconomic status can influence inflammation. These include beyond education, for example, exposure to xenobiotics and pollutants, junk food and infectious diseases, as well as psychosocial stress [31].

About food, an important turning point in the study of the relationship between diet, healthy ageing, and inflammation was the definition of the Diet Inflammatory Index (DII) which allows to quantify the pro or anti-inflammatory power of a specific food. Following the introduction of DII, it is possible to identify dietary regimens associated with an increased risk of metabolic disorders, CVDs, and cancer, and mutually healthy ageing and longevity [28].

As previously stated, oxidative stress plays an important role also in determining and maintaining the typical low-grade inflammation, in turn contributing to oxidative stress. Concerning Sicilian centenarians [7,32], the main product of the poly-unsaturated fatty acids peroxidation, malondialdehyde, is not significantly different between centenarians and controls of younger age. On the other hand, the range values of blood glutathione in these centenarians have been shown to be included in the value range of young people. Paraoxonase (PON) is an enzyme associated with high density lipoproteins, believed to protect against the oxidation (ox-) of low-density lipoproteins (LDL), so protecting from the risk of coronary artery disease. In Sicilian centenarians, PON units are not significantly different from those observed in young people. Interestingly, the value range of ox-LDL in these centenarians has been shown to be lower than that observed in young people It is seemingly puzzling that the total antioxidant capacity is lower in young people than in the other groups and that the highest values are observed in nonagenarians. It can be hypothesized that the eating habits of adults and older people, more adherent to MedDiet than young people (see above) could contribute to our observations [7,32]. Our results agree with most literature reporting decrease of oxidative stress in LLIs. In most studies, indeed, centenarians showed lower levels of lipid peroxides, and higher plasma levels of antioxidant vitamin E than older controls, suggesting that they may be better equipped to contrast oxidative stress [11].

Concerning inflammatory markers of Sicilian LLIs [7], we observed a not significant increase of neutrophil/lymphocyte (N/L) ratio that is an emerging inflammatory marker because it combines the predictive power of both decreased lymphocyte and increased neutrophil counts. In people aged 55 years and older, this ratio has been described to be associated with mortality [33]. Albumin and total proteins levels are decreased in LLIs in agreement with results obtained in several centenarian studies. Albumin has been used as a biochemical indicator of nutritional status, but decreased albumin levels are also a reflection of inflammatory status, as negative acute phase protein [34]. Significantly higher concentrations of CRP have been observed in Sicilian LLIs as compared to their controls, although the increase was not significant in centenarians, in the present study, as well as in several other reports [34]. The significant iron serum level decrease observed in Sicilian LLIs is not surprising because levels of circulating hepcidin, elevated in response to inflammation, are responsible for changes in iron metabolism that results in systemic iron depletion, although another contributory factor might be the scarcity of iron content in the diet [35]. In addition, Sicilian LLIs have been shown to present the kynurenine/tryptophan (Kyn/Trp) ratio higher than in all other age-groups [7]. The Kyn/Trp ratio has been suggested to represent a valuable marker for the rate of inflamm-ageing [36].

Is inflamm-ageing compatible with longevity, *i.e*., 100 years of age or more? As just demonstrated, the answer is positive and apparently paradoxical, since, even if centenarians may have an increased level of inflammatory mediators in comparison to older subjects and they are very frail, they should have high level of anti-inflammatory molecules together with protective genotypes.

Accordingly, LLIs belonging to this cohort displayed an increased enzymatic activity of the extracellular proteinase matrix metalloproteinase 2 known to regulate intercellular communication, including inflammation [37].

More interestingly are the data concerning the profiles of circulating microRNAs (miRNA) that appear to be related to chronological age [38]. Variations in plasmatic levels of mir-146a-5p, mir-126–3p, and mir-21–5p, three miRNAs involved in pathways related to inflammation, senescence, and carcinogenesis, seem to be characteristic of the longevity phenotype. Another ageing-associated microRNA is mir-181a. This miRNA is involved in the control of innate immunity and inflammation and its low levels are correlated to higher risk of coronary artery disease. These suggestions have been strengthened by two our previous studies. Recently [32], we have reported a case of a female supercentenarian in seemingly good health despite some laboratory signs of atrophic gastritis and a chronic status of inflammation. Her high level of plasmatic miR-181a suggested that the anti-inflammatory effects of this miRNA has conferred, to this subject protection against tissue damage. Previously we reported the exceptional case of two sisters, semi and supercentenarian [39], showing plasmatic levels of mir-146a-5p, mir-126–3p, and mir-21–5p comparable to those measured in young (24–39 years) and middle-aged individuals (50–64 years), rather than those found in older subjects (66–84 years). It is intriguing that all the three miRNAs are induced by endothelial dysfunctions and that supercentenarians have been claimed to markedly delay and even escape clinical expression of vascular disease toward the end of their exceptionally long lives. Moreover, the three microRNA plasmatic levels observed in sisters suggest that the two subjects were experiencing healthy ageing conditions relatively to age-related diseases. These observations on the control of inflammation and age-related disease development have been confirmed by the results of a recent paper we have performed in a cohort of Sicilians donors from young to supercentenarians. The circulating levels of four circulating miRNAs including miR-146a-5p, miR-126–3p, miR-21–5p, and miR-181a-5p, involved in several pathways related to inflammation, and endothelial cell senescence (ECs) were determined in 78 healthy Sicilians aged between 22 and 111 years. Contextually, extracellular miRna levels were measured in human ECs in vitro model, undergoing senescence. We found that the levels of the four miRNAs, using *ex vivo* and in vitro models, progressively increase with age, apart from ultra-centenarians that showed levels comparable to those measured in young individuals. Our results [40] contribute to the development of knowledge regarding the identification of miRNAs as biomarkers of successful and unsuccessful ageing. Indeed, they might have diagnostic/ prognostic relevance for age-related diseases. That, then, suggests the potentiality of miRNA levels restoration or the use of their mimics as therapeutic agents for age-related inflammatory diseases.

Also, anti-inflammatory genotypes may play a role [12]. In fact, male Sicilian centenarians, recruited in a precedent survey with Southern Italy centenarians, showed a higher frequency of the anti-inflammatory alleles of CC chemokine receptor 5, 5-lipoxygenase, cyclo-oxygenase 2, Toll-like-receptor-4 and cytokine genes, so favouring the control of inflamm-ageing and the onset of age-related diseases [41,42].

As previously described, age-related diseases are characterized by common background in which inflamm-ageing and oxidative stress play the major causative role. Therefore, the preventive measures should be early and complex, resulting in the lowering of pro-inflammatory state in the older, and so in complex protection from the age-related diseases [43–45].

This goal can be achieved by: i) Early poly-vaccination against pathogens known to recur or reinfect leading to inflammatory response; if a chronic infection is already present (cytomegalovirus as an example), it should be treated with the use of antiviral drugs, antibiotics, anti-mycotic to decrease the pathogen load and the amount of inflammatory response [44,46]; ii) Reasonable and personalized use of anti-inflammatory treatment even in healthy older exhibiting elevated levels of proinflammatory cytokines with statins and non-steroidal anti-inflammatory drugs [47]; iii) Preventing and curing microbiome dysbiosis with the use of pre- and probiotics since optimal gut microbiota plays a role as anti-inflammatory agent [48], iv) Preventing accumulation of senescent cells and disrupting these already present with the use of senolytics drugs as reported below.

Stimulation of cell death by using pharmacologically active small molecules, senolytic drugs, should represent a promising anti-inflammatory treatment and a more general anti-ageing treatment since senescent cells contribute to tissue dysfunction [49]. As discussed by Paez-Ribes et al. [50], different strategies can be potentially implemented. The use of apoptosis-inducing drugs that inhibit of pro-survival pathways represents the leading approach. In fact, inhibitors of the BCL-2 cell death regulator family of proteins can induce selective apoptosis of senescent cells. The dampening of the expression of SASP factors by molecules that interfere with transcriptional factors involved in the secretion of proinflammatory mediators has also proven beneficial in some settings. Then, genetic, and epigenetic manipulation of cells, including the induction of reprogramming, have been proposed as a means of bypassing or reverting the state of cellular senescence. Finally, the activation of the immune system against senescent cells to stimulate their clearance by enhancing the cytotoxic activity of NK against senescent cells, and manipulating the humoral innate immunity with the use of antibodies against receptors, represents another possibility. At this regard, recently it was reported that chimeric antigen receptor (CAR) T cells can be redirected to target senescent cells in animal models. CAR T cells that specifically target senescent cells could be a novel senolytic treatment strategy for senescence-associated diseases. In mice, senescence-targeted CAR T cells extended survival in lung cancer and improved liver fibrosis [51].

However, as Sicilian centenarians show, the most efficient interventions would seem to be the multi-modal lifelong lifestyle interventions. As discussed by Accardi et al. [6], that includes: i) The anti-inflammatory Mediterranean Diet characterized by abundance of fruit and vegetables rich in phyto-chemicals with anti-oxidant properties [52], and a limited intake of meat and of refined sugars; ii) physical exercise since exercise-deprivation induces a cluster of physiological abnormalities, like metabolic syndrome (such as insulin resistance, impaired glucose uptake and hyperlipidaemia); iii) satisfactory social interactions with a healthy psychological approach to mitigate life stress.

## 3. Conclusions

Population ageing has great social and economic consequences. As previously stated, ageing of population is a great achievement of humanity, but it also represents a challenge that Western world is currently facing, as ageing is associated with increased susceptibility to many diseases such as CVDs, cancer, T2D, Alzheimer and Parkinson diseases. Therefore, it is necessary to fully understand the mechanisms of successful ageing to prevent the harmful aspects of ageing. As discussed in this review, the study of centenarians is a fine model for trying to achieve this. On this basis, anti-ageing strategies aimed not to rejuvenate but to slow ageing and to delay or avoid the onset of age-related diseases have been discussed, hence people will be able to substantially slow down the ageing process, extending productive, youthful lives.

Successful ageing is inherently a highly plastic trait that responds to extensive lifestyle changes: exercise, eating habits, living conditions, and nutritional interventions as demonstrated by the following study. Analysed data on morbidity and mortality patterns of CVDs among 34 million Americans from 1999 to 2000 demonstrated that hospitalizations due to heart attack and stroke have reduced to 38% and 34%, respectively. The Authors attributed this dramatic success to lifestyle changes, besides better treatment, and preventive measures. Thus, both recommended and self-motivated lifestyle changes to reduce cardiovascular disease risks, must have also contributed to the overall health and longevity of at least a fraction of the U.S. population [53,54].

## Supplementary Information
