# Supplementary material for: Lessons from Sicilian Centenarians for Anti-Ageing Medicine. The Oxi-Inflammatory Status
Source: Transl Med UniSa. 2022 Sep 26;24(2):16–23. doi: 10.37825/2239-9754.1036 (PMC9673988; doi:10.37825/2239-9754.1036)
Supplement: Supplementary file 1 [file TMED-24-02-016-s001.pdf]

Translational Medicine @ UniSa

Manuscript 1036

Lessons from Sicilian centenarians for anti-ageing medicine. The Oxi-inflammatory status

Author #1

Author #2

Author #3

Author #4

Author #5

See next page for additional authors

Follow this and additional works at: https://tmj.unisa.it/journal

Part of the Health Communication Commons, Life Sciences Commons, and the Medicine and Health Sciences Commons

Lessons from Sicilian centenarians for anti-ageing medicine. The Oxi-inflammatory status

Authors

Author #1, Author #2, Author #3, Author #4, Author #5, and Author #6

REVIEW

Lessons from Sicilian Centenarians for Anti-Ageing Medicine. The Oxi-Inflammatory Status

Q3 Calogero Caruso*, Giulia Accardi, Anna Aiello, Anna Calabrò, Mattia E. Ligotti, Giuseppina Candore

Laboratory of Immunopathology and Immunosenescence, Department of Biomedicine, Neuroscience and Advanced Diagnostics, University of Palermo, Palermo, Italy

Abstract

Population ageing is a great achievement of humanity, but it also represents a challenge that the Western world is currently facing, as ageing is associated with increased susceptibility to age-related inflammatory diseases. Therefore, it is necessary to fully understand the mechanisms of healthy ageing to prevent the harmful aspects of ageing. The study of long living ndividuals (LLIs) is a great model for trying to achieve this goal. Accordingly, the oxy-inflammatory status of Sicilian LLIs was reviewed in the present paper. Based on the reported data, anti-inflammatory and anti-oxidative stress strategies have been discussed, useful for delaying or avoiding the onset of age-related diseases, thus favouring a healthy ageing process.

Keywords: Centenarians, Inflammation, Longevity, Oxidative stress

1. Introduction

People around the world are living longer. In addition, the pace of ageing of the world population is also increasing [1]. The increasing mean lifespan of the population is a big success of humanity, but also poses a challenge that industrialized countries are currently facing because the increase in lifespan does not coincide with the increase in the duration of health, health-span, *i.e.*, the period of life free from serious chronic diseases and disabilities [2]. Therefore, improving the quality of life of oldest people is becoming a priority due to the continuous increase in the number of this population who is at risk of frailty. This makes the studies of the processes involved in healthy ageing and longevity of great importance. The identification of the factors that predispose to a long and healthy life is therefore of enormous interest for translational medicine. This means understanding why some individuals, namely the centenarians, have escaped neonatal mortality, infectious diseases in the pre-antibiotic era, and the fatal outcomes of age-related diseases, thus living more than 100 years. The knowledge born from this approach could allow modulating the ageing rate by providing valuable information on lifestyle to achieve healthy ageing [3–5].

In the last twelve years, we have surveyed the population of some Sicilian small towns and villages, characterized by a high rate of centenarians to investigate the mechanisms involved in longevity [6–10]. Considering the key role played by inflammation and oxidative stress in mechanisms of age-related diseases [11], we report data related to the oxi-inflammatory status of a homogeneous population of Sicilian centenarians and nonagenarians (log-living individuals, LLIs) studied together with young people, adults, and older adults to have an adequate and matched number of controls. Data are summarized and discussed according to the available literature, for their possible implication for the prevention and/or treatment of age-related diseases.

Received 6 June 2022; accepted 26 August 2022.
Available online ■ ■ ■

* Corresponding author at:
E-mail address: calogero.caruso@unipa.it (C. Caruso).

https://doi.org/10.37825/2239-9754.1036
2239-9754/© 2022 Università di Salerno. This is an open access article under the CC BY 2.5 license (https://creativecommons.org/licenses/by/2.5/).

2. Oxi-inflammatory status of Sicilian LLIs

Inflammation is defined as a localized response with systemic consequences, elicited by trauma or infection, which helps to destroy, reduce, or sequester both the harmful agent and the wounded tissue, so supporting survival by fighting off pathogenic microbes and healing injuries. Therefore, the inflammation process is not *per se* a negative phenomenon; it is the response of the innate immune system to pathogenic viruses or bacteria. However, older organisms tend to develop a pro-inflammatory status characterized by high levels of pro-inflammatory markers in cells, tissues, and blood, a condition called inflamm-ageing. In fact, ageing is accompanied by chronic low grade inflammatory state. This chronic inflammation is instrumental in driving the ageing process in general, and contributes to the development of age-related diseases. In older people, the tissues have high levels of pro-inflammatory cytokines, which are known to interfere with anabolic signalling, thus contributing to the development of sarcopenia. During the life-history, low-grade inflammation, develops gradually and contributes to the pathogenesis of a range of age-related diseases as cardiovascular diseases (CDVs), and Type-2 diabetes (T2D), cancer, and neurodegeneration as well as frailty and sarcopenia (a far from exhaustive list is shown in Fig. 1). Therefore, this backdrop of low-grade inflammation contributes significantly to mortality risk of older people and has several sources, as discussed below [12−15].

Oxidative stress is an oxidative condition, resulting from an imbalance between oxidant and antioxidant factors in favour of the former ones, capable of damaging DNA, lipids, and proteins with consequent tissue damage and acceleration of ageing process. With advancing age, the concentrations of free radical increase because of the age-related functional decline in the electron transport chain and changes in the gene expression profile as well as by the decline of antioxidant systems [16]. The excess of free radicals interacts with lipids, proteins, and DNA, altering the normal physiological functions of cells. This ability allows to understand why oxidative stress is involved in the pathophysiological mechanisms of age-related diseases. In addition, the oxidative stress is thought to be closely related to inflamm-ageing [17]. The increase of free radicals is correlated to damage-associated molecular pattern (DAMPs) release, endogenous nuclear or cytosolic molecules released from injured and dying cells. DAMPs are able to activate inflammasome, through specific receptors, promoting the production of pro-inflammatory cytokines. In turn, these cytokines activate inflammatory cells, further potentiating free radicals production, in a vicious cycle [11].

More in general, cellular injury can release DAMPs activating innate immunity. So, cell debris caused by inappropriate cell destruction and clearance during the ageing process can trigger the innate immune system, which sets the scene for persistent inflammation. Cell debris accumulate with age because of both increased production and impaired elimination by defective autophagy [18,19].

However, the most important causes of inflamma-ageing are represented by cellular senescence and pathogen burden, both linked to the ageing of immune system, *i.e.,* immunosenescence; in turn, inflamm-ageing contributes to immunosenescence [20,21].

Cells are driven into a senescent, non-dividing state by several factors, including telomere shortening, DNA damage, and inflammatory cytokines. These all result in the activation of transcription factor p53 which is involved in a variety of processes including DNA repair and apoptosis, but most notably results in expression of cyclin-dependent kinase inhibitor p21, which together with activation of p16, is the major pathways in the induction of senescence. The immune system clears away these damaged cells; however, in older people immunosenescence is responsible for a less efficiency of the clearing of these cells declines, hence increasing numbers linger in tissues and secrete an inflammatory cocktail of cytokines known as the senescence-associated secretory phenotype (SASP) [21,22].

It has been suggested that the reductio[illegible] in lifetime exposure to infectious diseases and other sources of inflammation contributes to the historical decline in old-age mortality, strengthening the suggestion that long-life pathogen burden is a very important factor for age-related inflammation. Persistent antigenic challenges lead to a poor response to newly encountered microbial antigens, as well as to a shift in the immune system toward an inflammatory T helper profile. Late-stage memory effector T-cells contribute to the pro-inflammatory state in older people as producers of pro-inflammatory cytokines. In addition, the long-term chronic microbial burden induces progressive activation of macrophages, hence contributing to the chronic state of low-grade inflammation. Accordingly, some studies have linked an individual exposure to past infection to levels of chronic inflammation and to

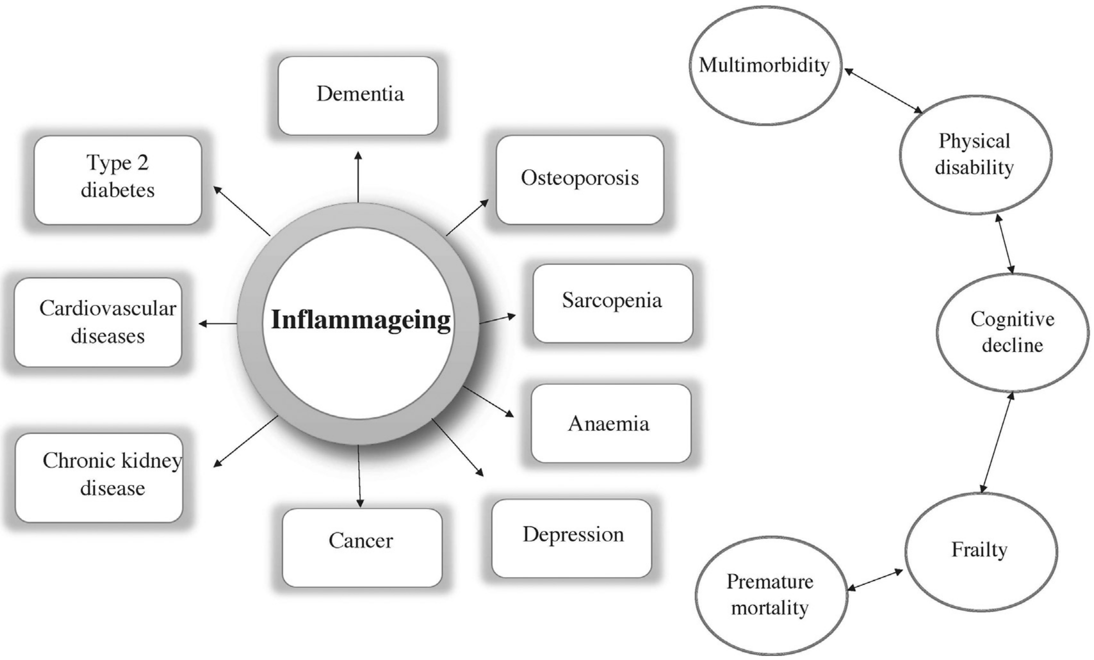

Fig. 1. Cardiovascular diseases, chronic kidney disease, cancer, depression, anaemia, sarcopenia, osteoporosis, and dementia are shown in the figure as example of inflammatory age-related diseases, since inflamm-ageing contributes to the development of these diseases in older people. Moreover, elevated blood levels of pro-inflammatory markers are a powerful risk facor for multimorbidity that, with inflamm-ageing, is a strong risk factor for physical and cognitive disability, frailty, and premature mortality. References in the text.

increased risk of heart attack, stroke, and cancer [14,15,20,23].

On the other hand, bacteria have developed an intimate relationship with animals, colonizing specific body sites at the interface with the body exterior and invaginations, hence constituting an integrated meta-organism with functional integration conferring significant advantages to animals and bacteria. The immune system of the host coevolved with the microbiota to develop complex mechanisms to recognize and destroy invading microbes, while preserving its own bacteria. However, the oral and gut mucosa barriers that protect against bacterial invasion begin to both decline in effectiveness and break down as human beings age. Periodontal disease has been shown to shown to contribute to inflamm-ageing by generating chronic, low-grade inflammation. In the gut, the microbiome shows an increasing decline of diversity with age. Composition and diversity of the microbiota change according to development and ageing and contribute to health and fitness by modulating the immune system response and inflamm-ageing and vice versa [14,24–26]. Bian et al. [27], collected and examined the gut microbiota of a cross-sectional cohort of more than 1000 very healthy Chinese individuals who spanned ages from 3 to over 100 years, showing that the healthiest aged people had gut microbiomes like much younger people; in other words, they maintained diversity and thus lower levels of inflammation.

Closely related to inflamm-ageing is metaflammation, the low-grade, chronic inflammation orchestrated by metabolic cells in response to excess nutrients and energy. An excess of macronutrients introduced with the diet determines an increased circulation of fatty acids and obesity. That leads to the stimulation of macrophages and the same adipocytes, triggering signal transduction pathways involved in the pro-inflammatory status. In fact, in subjects affected by visceral obesity increased levels of pro-inflammatory cytokines and decreased adiponectin levels are observed. That triggers an increased production of free radicals [28]. Thus, pro-inflammatory activation of macrophages in metabolic tissues is critically important in the induction of obesity-induced metaflammation and it has been demonstrated that the soluble mannose receptor plays a direct functional role in both macrophage activation and metaflammation [29]. In turn, the pro-inflammatory status induced by metaflammation as well as inflamm-ageing leads to cellular damage and senescence, so worsening inflamm-ageing [28].

In a recent multi-cohort study [30], then, using data from several European countries, it was

observed that a disadvantageous socioeconomic status at any stage of life was associated with an increase in inflammation assessed using serum levels of C-reactive protein (CRP), an acute phase reactant that responds rapidly to tissue injury, infection, and inflammation. In general, known behavioural factors (alcohol consumption, smoking habit, and sedentary lifestyle) and body mass index (BMI) explained in part, but not completely, the relationship between socioeconomic status and inflammation. A subsequent analysis indicated that low-educated participants had higher CRP levels regardless of socioeconomic status in early life or adulthood, as well as behavioural factors and BMI. The persistence of a significant association between low level of education and a high level of CRP in adulthood indicate that the level of education may be an upstream risk factor for high inflammation. These findings suggest that there are other pathways through which socioeconomic status can influence inflammation. These include beyond education, for example, exposure to xenobiotics and pollutants, junk food and infectious diseases, as well as psychosocial stress [31].

About food, an important turning point in the study of the relationship between diet, healthy ageing, and inflammation was the definition of the Diet Inflammatory Index (DII) which allows to quantify the pro or anti-inflammatory power of a specific food. Following the introduction of DII, it is possible to identify dietary regimens associated with an increased risk of metabolic disorders, CVDs, and cancer, and mutually healthy ageing and longevity [28].

As previously stated, oxidative stress plays an important role also in determining and maintaining the typical low-grade inflammation, in turn contributing to oxidative stress. Concerning Sicilian centenarians [7,32], the main product of the polyunsaturated fatty acids peroxidation, malondialdehyde, is not significantly different between centenarians and controls of younger age. On the other hand, the range values of blood glutathione in these centenarians have been shown to be included in the value range of young people. Paraoxonase (PON) is an enzyme associated with high density lipoproteins, believed to protect against the oxidation (ox-) of low-density lipoproteins (LDL), so protecting from the risk of coronary artery disease. In Sicilian centenarians, PON units are not significantly different from those observed in young people. Interestingly, the value range of ox-LDL in these centenarians has been shown to be lower than that observed in young people It is seemingly puzzling that the total antioxidant capacity is lower in young people than in the other groups and that the highest values are observed in nonagenarians. It can be hypothesized that the eating habits of adults and older people, more adherent to MedDiet than young people (see above) could contribute to our observations [7,32]. Our results agree with most literature reporting decrease of oxidative stress in LLIs. In most studies, indeed, centenarians showed lower levels of lipid peroxides, and higher plasma levels of antioxidant vitamin E than older controls, suggesting that they may be better equipped to contrast oxidative stress [11].

Concerning inflammatory markers of Sicilian LLIs [7], we observed a not significant increase of neutrophil/lymphocyte (N/L) ratio that is an emerging inflammatory marker because it combines the predictive power of both decreased lymphocyte and increased neutrophil counts. In people aged 55 years and older, this ratio has been described to be associated with mortality [33]. Albumin and total proteins levels are decreased in LLIs in agreement with results obtained in several centenarian studies. Albumin has been used as a biochemical indicator of nutritional status, but decreased albumin levels are also a reflection of inflammatory status, as negative acute phase protein [34]. Significantly higher concentrations of CRP have been observed in Sicilian LLIs as compared to their controls, although the increase was not significant in centenarians, in the present study, as well as in several other reports [34]. The significant iron serum level decrease observed in Sicilian LLIs is not surprising because levels of circulating hepcidin, elevated in response to inflammation, are responsible for changes in iron metabolism that results in systemic iron depletion, although another contributory factor might be the scarcity of iron content in the diet [35]. In addition, Sicilian LLIs have been shown to present the kynurenine/tryptophan (Kyn/Trp) ratio higher than in all other age-groups [7]. The Kyn/Trp ratio has been suggested to represent a valuable marker for the rate of inflamm-ageing [36].

Is inflamm-ageing compatible with longevity, *i.e.*, 100 years of age or more? As just demonstrated, the answer is positive and apparently paradoxical, since, even if centenarians may have an increased level of inflammatory mediators in comparison to older subjects and they are very frail, they should have high level of anti-inflammatory molecules together with protective genotypes.

Accordingly, LLIs belonging to this cohort displayed an increased enzymatic activity of the extracellular proteinase matrix metalloproteinase 2 known to regulate intercellular communication, including inflammation [37].

More interestingly are the data concerning the profiles of circulating microRNAs (miRNA) that appear to be related to chronological age [38]. Variations in plasmatic levels of mir-146a-5p, mir-126–3p, and mir-21–5p, three miRNAs involved in pathways related to inflammation, senescence, and carcinogenesis, seem to be characteristic of the longevity phenotype. Another ageing-associated microRNA is mir-181a. This miRNA is involved in the control of innate immunity and inflammation and its low levels are correlated to higher risk of coronary artery disease. These suggestions have been strengthened by two our previous studies. Recently [32], we have reported a case of a female supercentenarian in seemingly good health despite some laboratory signs of atrophic gastritis and a chronic status of inflammation. Her high level of plasmatic miR-181a suggested that the anti-inflammatory effects of this miRNA has conferred, to this subject protection against tissue damage. Previously we reported the exceptional case of two sisters, semi and supercentenarian [39], showing plasmatic levels of mir-146a-5p, mir-126–3p, and mir-21–5p comparable to those measured in young (24–39 years) and middle-aged individuals (50–64 years), rather than those found in older subjects (66–84 years). It is intriguing that all the three miRNAs are induced by endothelial dysfunctions and that supercentenarians have been claimed to markedly delay and even escape clinical expression of vascular disease toward the end of their exceptionally long lives. Moreover, the three microRNA plasmatic levels observed in sisters suggest that the two subjects were experiencing healthy ageing conditions relatively to age-related diseases. These observations on the control of inflammation and age-related disease development have been confirmed by the results of a recent paper we have performed in a cohort of Sicilians donors from young to supercentenarians. The circulating levels of four circulating miRNAs including miR-146a-5p, miR-126–3p, miR-21–5p, and miR-181a-5p, involved in several pathways related to inflammation, and endothelial cell senescence (ECs) were determined in 78 healthy Sicilians aged between 22 and 111 years. Contextually, extracellular miRna levels were measured in human ECs in vitro model, undergoing senescence. We found that the levels of the four miRNAs, using *ex vivo* and in vitro models, progressively increase with age, apart from ultra-centenarians that showed levels comparable to those measured in young individuals. Our results [40] contribute to the development of knowledge regarding the identification of miRNAs as biomarkers of successful and unsuccessful ageing. Indeed, they might have diagnostic/prognostic relevance for age-related diseases. That, then, suggests the potentiality of miRNA levels restoration or the use of their mimics as therapeutic agents for age-related inflammatory diseases.

Also, anti-inflammatory genotypes may play a role [12]. In fact, male Sicilian centenarians, recruited in a precedent survey with Southern Italy centenarians, showed a higher frequency of the anti-inflammatory alleles of CC chemokine receptor 5, 5-lipoxygenase, cyclo-oxygenase 2, Toll-like-receptor-4 and cytokine genes, so favouring the control of inflamm-ageing and the onset of age-related diseases [41,42].

As previously described, age-related diseases are characterized by common background in which inflamm-ageing and oxidative stress play the major causative role. Therefore, the preventive measures should be early and complex, resulting in the lowering of pro-inflammatory state in the older, and so in complex protection from the age-related diseases [43–45].

This goal can be achieved by: i) Early poly-vaccination against pathogens known to recur or reinfect leading to inflammatory response; if a chronic infection is already present (cytomegalovirus as an example), it should be treated with the use of antiviral drugs, antibiotics, anti-mycotic to decrease the pathogen load and the amount of inflammatory response [44,46]; ii) Reasonable and personalized use of anti-inflammatory treatment even in healthy older exhibiting elevated levels of proinflammatory cytokines with statins and non-steroidal anti-inflammatory drugs [47]; iii) Preventing and curing microbiome dysbiosis with the use of pre- and probiotics since optimal gut microbiota plays a role as anti-inflammatory agent [48], iv) Preventing accumulation of senescent cells and disrupting these already present with the use of senolytics drugs as reported below.

Stimulation of cell death by using pharmacologically active small molecules, senolytic drugs, should represent a promising anti-inflammatory treatment and a more general anti-ageing treatment since senescent cells contribute to tissue dysfunction [49]. As discussed by Paez-Ribes et al. [50], different strategies can be potentially implemented. The use of apoptosis-inducing drugs that inhibit of pro-survival pathways represents the leading approach. In fact, inhibitors of the BCL-2 cell death regulator family of proteins can induce selective apoptosis of senescent cells. The dampening of the expression of SASP factors by molecules that interfere with transcriptional factors involved in the secretion of pro-inflammatory mediators has also proven beneficial in some settings. Then, genetic, and epigenetic

manipulation of cells, including the induction of reprogramming, have been proposed as a means of bypassing or reverting the state of cellular senescence. Finally, the activation of the immune system against senescent cells to stimulate their clearance by enhancing the cytotoxic activity of NK against senescent cells, and manipulating the humoral innate immunity with the use of antibodies against receptors, represents another possibility. At this regard, recently it was reported that chimeric antigen receptor (CAR) T cells can be redirected to target senescent cells in animal models. CAR T cells that specifically target senescent cells could be a novel senolytic treatment strategy for senescence-associated diseases. In mice, senescence-targeted CAR T cells extended survival in lung cancer and improved liver fibrosis [51].

However, as Sicilian centenarians show, the most efficient interventions would seem to be the multimodal lifelong lifestyle interventions. As discussed by Accardi et al. [6], that includes: i) The anti-inflammatory Mediterranean Diet characterized by abundance of fruit and vegetables rich in phytochemicals with anti-oxidant properties [52], and a limited intake of meat and of refined sugars; ii) physical exercise since exercise-deprivation induces a cluster of physiological abnormalities, like metabolic syndrome (such as insulin resistance, impaired glucose uptake and hyperlipidaemia); iii) satisfactory social interactions with a healthy psychological approach to mitigate life stress.

3. Conclusions

Population ageing has great social and economic consequences. As previously stated, ageing of population is a great achievement of humanity, but it also represents a challenge that Western world is currently facing, as ageing is associated with increased susceptibility to many diseases such as CVDs, cancer, T2D, Alzheimer and Parkinson diseases. Therefore, it is necessary to fully understand the mechanisms of successful ageing to prevent the harmful aspects of ageing. As discussed in this review, the study of centenarians is a fine model for trying to achieve this. On this basis, anti-ageing strategies aimed not to rejuvenate but to slow ageing and to delay or avoid the onset of age-related diseases have been discussed, hence people will be able to substantially slow down the ageing process, extending productive, youthful lives.

Successful ageing is inherently a highly plastic trait that responds to extensive lifestyle changes: exercise, eating habits, living conditions, and nutritional interventions as demonstrated by the following study. Analysed data on morbidity and mortality patterns of CVDs among 34 million Americans from 1999 to 2000 demonstrated that hospitalizations due to heart attack and stroke have reduced to 38% and 34%, respectively. The Authors attributed this dramatic success to lifestyle changes, besides better treatment, and preventive measures. Thus, both recommended and self-motivated lifestyle changes to reduce cardiovascular disease risks, must have also contributed to the overall health and longevity of at least a fraction of the U.S. population [53,54].

Acknowledgements Q1

Original work of Laboratory of Immunopathology and Immunosenescence is supported by Italian Ministry of University (PRIN: progetti di ricerca di rilevante interesse nazionale—Bando 2015 Prot 20157ATSLF Discovery of molecular and genetic/epigenetic signatures underlying resistance to age-related diseases and comorbidities) and by European Commission (Improved Vaccination Strategies for Older Adults granted - Horizon 2020 ID 848166). Q2

References

[1] Who. https://www.who.int/news-room/fact-sheets/detail/ageing-and-health (accessed July 31, 2021).
[2] Crimmins EM. Lifespan and healthspan: past, present, and promise. Gerontol 2015;55:901–11. https://doi.org/10.1093/geront/gnv130.
[3] Accardi G, Aiello A, Vasto S, Caruso C. Chance and causality in ageing and longevity. In: Caruso C, editor. Centenarians. Cham: Springer; 2019. https://doi.org/10.1007/978-3-030-20762-5_1.
[4] Caruso C, Passarino G, Puca A, Scapagnini G. Positive biology": the centenarian lesson. Immun Ageing 2012;9:5. https://doi.org/10.1186/1742-4933-9-5.
[5] Caruso C, Puca AA. Special issue "Centenarians-A model to study the molecular basis of lifespan and healthspan. Int J Mol Sci 2021;22:2044. https://doi.org/10.3390/ijms22042044.
[6] Accardi G, Aprile S, Candore G, Caruso C, Cusimano R, Cristaldi L, et al. Genotypic and phenotypic aspects of longevity: results from a Sicilian survey and implication for the prevention and treatment of age-related diseases. Curr Pharmaceut Des 2019;25:228–35. https://doi.org/10.2174/1381612825666190313115233.
[7] Aiello A, Accardi G, Aprile S, Caldarella R, Carru C, Ciaccio M, et al. Age and gender-related variations of molecular and phenotypic parameters in A cohort of Sicilian population: from young to centenarians. Aging and disease 2021;12:1773–93. https://doi.org/10.14336/AD.2021.0226.
[8] Ligotti ME, Aiello A, Accardi G, Aprile S, Bonura F, Bulati M, et al. Analysis of T and NK cell subsets in the Sicilian population from young to supercentenarian: the role of age and gender. Clin Exp Immunol 2022;205:198–212. https://doi.org/10.1111/cei.13606.
[9] Vasto S, Scapagnini G, Rizzo C, Monastero R, Marchese A, Caruso C. Mediterranean diet and longevity in Sicily: survey in a Sicani Mountains population. Rejuvenation Res 2012;15:184–8. https://doi.org/10.1089/rej.2011.1280.
[10] Vasto S, Rizzo C, Caruso C. Centenarians and diet: what they eat in the Western part of Sicily. Immun Ageing 2012;9:10. https://doi.org/10.1186/1742-4933-9-10.

[11] Accardi G, Ligotti ME, Candore G. Phenotypic aspects of longevity. In: Caruso C, editor. Centenarians. Cham: Springer; 2019. https://doi.org/10.1007/978-3-030-20762-5_2.
[12] Ferrucci L, Fabbri E. Inflammageing: chronic inflammation in ageing, cardiovascular disease, and frailty. Nat Rev Cardiol 2018;15:505–22. https://doi.org/10.1038/s41569-018-0064-2.
[13] Franceschi C, Campisi J. Chronic inflammation (inflammaging) and its potential contribution to age-associated diseases. J Gerontol A Biol Sci Med Sci 2014;69 S1:S4–9. https://doi.org/10.1093/gerona/glu057.
[14] Licastro F, Candore G, Lio D, Porcellini E, Colonna-Romano G, Franceschi C, et al. Innate immunity and inflammation in ageing: a key for understanding age-related diseases. Immun Ageing 2005;2:8. https://doi.org/10.1186/1742-4933-2-8.
[15] Vasto S, Candore G, Balistreri CR, Caruso M, Colonna-Romano G, Grimaldi MP, et al. Inflammatory networks in ageing, age-related diseases and longevity. Mech Ageing Dev 2007;128:83–91. https://doi.org/10.1016/j.mad.2006.11.015.
[16] Caruso C, Accardi G, Ligotti ME, Vasto S, Candore G. Pathobiology of aging: an introduction to age-related diseases. In: Caruso C, Candore G, editors. Human aging. Academic Press; 2021. https://doi.org/10.1016/B978-0-12-822569-1.00010-X.
[17] De la Fuente M, Miquel J. An update of the oxidation-inflammation theory of aging: the involvement of the immune system in oxi-inflamm-aging. Curr Pharmaceut Des 2009;15:3003–26. https://doi.org/10.2174/138161209789058110.
[18] Franceschi C, Garagnani P, Vitale G, Capri M, Salvioli S. Inflammaging and 'garb-aging. Trends Endocrinol Metabol 2017;28:199–212. https://doi.org/10.1016/j.tem.2016.09.005.
[19] Zhang Q, Raoof M, Chen Y, Sumi Y, Sursal T, Junger W, et al. Circulating mitochondrial DAMPs cause inflammatory responses to injury. Nature 2010;464:104–7. https://doi.org/10.1038/nature08780.
[20] Aiello A, Farzaneh F, Candore G, Caruso C, Davinelli S, Gambino CM, et al. Immunosenescence and its hallmarks: how to oppose aging strategically? A review of potential options for therapeutic intervention. Front Immunol 2019;10:2247. https://doi.org/10.3389/fimmu.2019.02247.
[21] Yousefzadeh MJ, Flores RR, Zhu Y, Schmiechen ZC, Brooks RW, Trussoni CE, et al. An aged immune system drives senescence and ageing of solid organs. Nature 2021;594:100–5. https://doi.org/10.1038/s41586-021-03547-7.
[22] de Magalhães JP, Passos JF. Stress, cell senescence and organismal ageing. Mech Ageing Dev 2018;170:2–9. https://doi.org/10.1016/j.mad.2017.07.001.
[23] Finch CE, Crimmins EM. Inflammatory exposure and historical changes in human life-spans. Science 2004;305:1736–9. https://doi.org/10.1126/science.1092556.
[24] Campisi G, Chiappelli M, De Martinis M, Franco V, Ginaldi L, Guiglia R, et al. Pathophysiology of age-related diseases. Immun Ageing 2009;6:12. https://doi.org/10.1186/1742-4933-6-12.
[25] Santoro A, Zhao J, Wu L, Carru C, Biagi E, Franceschi C. Microbiomes other than the gut: inflammaging and age-related diseases. Semin Immunopathol 2020;42:589–605. https://doi.org/10.1007/s00281-020-00814-z.
[26] Wang J, Chen WD, Wang YD. The relationship between gut microbiota and inflammatory diseases: the role of macrophages. Front Microbiol 2020;11:1065. https://doi.org/10.3389/fmicb.2020.01065.
[27] Bian G, Gloor GB, Gong A, Jia C, Zhang W, Hu J, et al. The gut microbiota of healthy aged Chinese is similar to that of the healthy young. mSphere 2017;2. https://doi.org/10.1128/mSphere.00327-17. e00327-17.
[28] Falzone L, Libra M, Polesel J. Dietary inflammatory index in ageing and longevity. In: Caruso C, editor. Centenarians. Cham: Springer; 2019. https://doi.org/10.1007/978-3-030-20762-5_5.
[29] Embgenbroich M, van der Zande HJP, Hussaarts L, Schulte-Schrepping J, Pelgrom LR, García-Tardón N, et al. Soluble mannose receptor induces proinflammatory macrophage activation and metaflammation. Proc Natl Acad Sci U S A 2021;118:e2103304118. https://doi.org/10.1073/pnas.2103304118.
[30] Berger E, Castagné R, Chadeau-Hyam M, Bochud M, d'Errico A, Gandini M, et al. Multi-cohort study identifies social determinants of systemic inflammation over the life course. Nat Commun 2019;10:773. https://doi.org/10.1038/s41467-019-08732-x.
[31] Caruso C, Accardi G, Aiello A, Candore G. Ambiente, invecchiamento e longevità. In: Ferrara A, editor. Trattato italiano di Medicina d'Ambiente. Società Editrice Universo; 2021 [in Italian)].
[32] Aiello A, Accardi G, Aprile S, Caldarella R, Cammarata G, Carru C, et al. Pro-inflammatory status is not a limit for longevity: case report of a Sicilian centenarian. Aging Clin Exp Res 2021;33:1403–7. https://doi.org/10.1007/s40520-020-01628-7.
[33] Fest J, Ruiter TR, Groot Koerkamp B, Rizopoulos D, Ikram MA, van Eijck CHJ, et al. The neutrophil-to-lymphocyte ratio is associated with mortality in the general population: the Rotterdam Study. Eur J Epidemiol 2019;34:463–70. https://doi.org/10.1007/s10654-018-0472-y.
[34] Hausman DB, Fischer JG, Johnson MA. Protein, lipid, and hematological biomarkers in centenarians: definitions, interpretation and relationships with health. Maturitas 2012;71:205–12. https://doi.org/10.1016/j.maturitas.2011.12.002.
[35] Wawer AA, Jennings A, Fairweather-Tait SJ. Iron status in the elderly: a review of recent evidence. Mech Ageing Dev 2018;175:55–73. https://doi.org/10.1016/j.mad.2018.07.003.
[36] Sorgdrager FJH, Naudé PJW, Kema IP, Nollen EA, Deyn PP. Tryptophan metabolism in inflammaging: from biomarker to therapeutic target. Front Immunol 2019;10:2565. https://doi.org/10.3389/fimmu.2019.02565.
[37] Cancemi P, Aiello A, Accardi G, Caldarella R, Candore G, Caruso C, et al. The role of matrix metalloproteinases (MMP-2 and MMP-9) in ageing and longevity: focus on Sicilian long-living individuals (LLIs). Mediat Inflamm 2020;2020:8635158. https://doi.org/10.1155/2020/8635158.
[38] Cammarata G, Duro G, Chiara TD, Curto AL, Taverna S, Candore G. Circulating miRNAs in successful and unsuccessful aging. A mini-review. Curr Pharmaceut Des 2019;25:4150–3. https://doi.org/10.2174/1381612825666191119091644.
[39] Accardi G, Aiello A, Aprile S, Caldarella R, Cammarata G, Carru C, et al. The phenotypic characterization of the cammalleri sisters, an example of exceptional longevity. Rejuvenation Res 2020;23:476–84. https://doi.org/10.1089/rej.2019.2299.
[40] Accardi G, Bono F, Cammarata G, Aiello A, Herrero MT, Alessandro R, et al. miR-126-3p and miR-21-5p as hallmarks of bio-positive ageing; correlation analysis and machine learning prediction in young to ultra-centenarian Sicilian population. Cells 2022;11:1505. https://doi.org/10.3390/cells11091505.
[41] Balistreri CR, Candore G, Accardi G, Bova M, Buffa S, Bulati M, et al. Genetics of longevity. data from the studies on Sicilian centenarians. Immun Ageing 2012;9:8. https://doi.org/10.1186/1742-4933-9-8.
[42] Incalcaterra E, Accardi G, Balistreri CR, Caimi G, Candore G, Caruso M, et al. Pro-inflammatory genetic markers of atherosclerosis. Curr Atherosclerosis Rep 2013;15:329. https://doi.org/10.1007/s11883-013-0329-5.
[43] Caruso C, Candore G. Conclusions. Slowing aging and fighting age-related diseases, from bench to bedside? In: Caruso C, Candore G, editors. Human aging. Academic Press; 2021. https://doi.org/10.1016/B978-0-12-822569-1.00001-9.
[44] Witkowski JM, Bryl E, Fulop T. In: Caruso C, Candore G, editors. The role of inflammaging in the development of chronic diseases of older people. Human aging. Academic Press; 2021. p. 89–104. https://doi.org/10.1016/B978-0-12-822569-1.00014-7.

[45] Caruso C, Aiello A, Accardi G, Ciaglia E, Cattaneo M, Puca A. Genetic signatures of centenarians: implications for achieving successful aging. Curr Pharmaceut Des 2019; 25:4133–8. https://doi.org/10.2174/1381612825666191112094544.

[46] Aiello A, Accardi G, Candore G, Caruso C, Colomba C, Di Bona D, et al. Role of immunogenetics in the outcome of HCMV infection: implications for ageing. Int J Mol Sci 2019; 20:685. https://doi.org/10.3390/ijms20030685.

[47] Wongrakpanich S, Wongrakpanich A, Melhado K, Rangaswami J. A comprehensive review of non-steroidal anti-inflammatory drug use in the elderly. Aging Dis 2018;9: 143–50. https://doi.org/10.14336/AD.2017.0306.

[48] Azad MAK, Sarker M, Li T, Yin J. Probiotic species in the modulation of gut microbiota: an overview. BioMed Res Int 2018;2018:9478630. https://doi.org/10.1155/2018/9478630.

[49] Kirkland JL, Tchkonia T. Senolytic drugs: from discovery to translation. J Intern Med 2020;288:518–36. https://doi.org/10.1111/joim.13141.

[50] Paez-Ribes M, González-Gualda E, Doherty GJ, Muñoz-Espín D. Targeting senescent cells in translational medicine. EMBO Mol Med 2019;11:e10234. https://doi.org/10.15252/emmm.201810234.

[51] Amor C, Feucht J, Leibold J, Ho YJ, Zhu C, Alonso-Curbelo D, et al. Senolytic CAR T cells reverse senescence-associated pathologies. Nature 2020;583:127–32. https://doi.org/10.1038/s41586-020-2403-9.

[52] Alì S, Davinelli S, Accardi G, Aiello A, Caruso C, Duro G, et al. Healthy ageing and Mediterranean diet: a focus on hormetic phytochemicals. Mech Ageing Dev 2021 Dec;200: 111592. https://doi.org/10.1016/j.mad.2021.111592.

[53] Govindaraju D, Atzmon G, Barzilai N. Genetics, lifestyle and longevity: lessons from centenarians. Appl Transl Genom 2015;4:23–32. https://doi.org/10.1016/j.atg.2015.01.001.

[54] Krumholz HM, Normand SL, Wang Y. Trends in hospitalizations and outcomes for acute cardiovascular disease and stroke, 1999-2011. Circulation 2014;130:966–75. https://doi.org/10.1161/CIRCULATIONAHA.113.007787.

AUTHOR QUERY FORM

**Journal:** TMJ<br><br>**Article Number:** 1036	**Please share your corrections or revision and any artwork related corrections to Digital common site.**<br><br>**E-mail:** **publishing.services@cs.bepress.com**

Dear Author,

Please check your proof carefully and mark all corrections at the appropriate place in the proof (e.g., by using on-screen annotation in the PDF file) or compile them in a separate list. **It is crucial that you NOT make direct edits to the PDF using the editing tools as doing so could lead us to overlook your desired changes.** Note: if you opt to annotate the file with software other than Adobe Reader then please also highlight the appropriate place in the PDF file. To ensure fast publication of your paper please return your corrections within 48 hours.

Any queries or remarks that have arisen during the processing of your manuscript are listed below and highlighted by flags in the proof.

**Location in article**	**Query / Remark: Click on the Q link to find the query's location in text**<br>**Please insert your reply or correction at the corresponding line in the proof**
Q1	Please provide a conflict of interest statement. If there is no conflict of interest, state that.
Q2	Correctly acknowledging the primary funders and grant IDs of your research is important to ensure compliance with funder policies. We could not find any acknowledgement of funding sources in your text. Is this correct?
Q3	Please confirm that given names and surnames have been identified correctly and are presented in the desired order and please carefully verify the spelling of all authors' names.

Thank you for your assistance.
